# Stevens-Johnson syndrome/toxic epidermal necrolysis induced by tislelizumab: a case report and literature review

**DOI:** 10.3389/fimmu.2025.1689877

**Published:** 2025-11-19

**Authors:** Hongtao Yu, Yunqian Li, Xifang Qu, Junkai Zhu, Zhichao Liu, Zhen Mu

**Affiliations:** 1 Department of Graduate Studies, Shandong First Medical University & Shandong Academy of Medical Sciences, Jinan, China; 2 Department of Dermatology, the Second Affiliated Hospital of Shandong First Medical University, Tai’an, China; 3 Department of Dermatology, Qilu Hospital of Shandong University, Jinan, China; 4 Department of Dermatology, Tai’an 88 Hospital, Tai’an, China

**Keywords:** SJS/TEN, severe drug eruption, tislelizumab, PD-1 inhibitor, TNF-α inhibitor

## Abstract

Tislelizumab, a programmed cell death-1 inhibitor approved for multiple malignancies, may induce Stevens-Johnson syndrome/toxic epidermal necrolysis (SJS/TEN)—a rare but potentially fatal severe drug eruption—and early effective intervention is pivotal for reducing SJS/TEN-related mortality. We describe an elderly male with hepatic malignancy who developed progressive SJS/TEN, involving over 80% of his total body surface area, after his first tislelizumab infusion. Conventional treatment with systemic corticosteroids combined with intravenous immunoglobulin was ineffective in halting disease progression. Subsequently, adjunctive therapy with a tumor necrosis factor-α (TNF-α) inhibitor and hemoperfusion was initiated, leading to the patient’s eventual recovery. Managing SJS/TEN in patients with advanced malignancies poses substantial challenges, given the life-threatening nature of both entities. To our knowledge, this is the first reported case of using a TNF-α inhibitor for treating tislelizumab-induced SJS/TEN. This case highlights a novel, cost-effective, and well-tolerated therapeutic strategy that yielded favorable outcomes.

## Introduction

Stevens-Johnson syndrome/toxic epidermal necrolysis (SJS/TEN) is a severe drug eruption characterized by extensive epidermal necrosis and desquamation, carrying high mortality rates (1, 2). Severe cases may involve organ damage, and mortality is frequently attributable to secondary complications such as pulmonary infections, hepatorenal failure, or electrolyte imbalances. Common allergenic drugs include antibiotics, antiepileptics, allopurinol, and antituberculosis agents (3).

Tislelizumab, a programmed cell death-1 (PD-1) inhibitor, is approved in China for the treatment of classical Hodgkin lymphoma, lung cancer, hepatocellular carcinoma, and other malignancies (4). Tislelizumab-induced SJS/TEN is comparatively rare. Herein, we describe a case of tislelizumab-induced SJS/TEN successfully managed with a tumor necrosis factor-α (TNF-α) inhibitor plus hemoperfusion, which, to our knowledge, is the first report detailing the successful use of a TNF-α inhibitor for this specific condition.

## Case presentation

A 65-year-old male presented to our hospital on June 22, 2025, with a 10-day history of generalized painful, pruritic erythema. Physical examination revealed widespread erythema of varying sizes across the entire body surface, non-blanching under pressure, with more than 70% of his total body surface area (TBSA) affected. Some erythematous lesions coalesced into patches, accompanied by scattered vesicles and bullae with thin walls and clear contents. Localized erosions and oral mucosal involvement were observed. Notably, approximately 5% of his TBSA was affected by skin detachment or mucosal erosion, whereas over 70% of his TBSA was involved by erythema. Initially, the SCORTEN (severity-of-illness score for toxic epidermal necrolysis) was 2, corresponding to a predicted mortality rate of 12.1%. Approximately 40 days prior to presentation, he had been diagnosed with primary liver malignancy, bilateral lung metastases, and chronic hepatitis B virus (HBV) infection. He received a 200-mg dose of tislelizumab on May 9, 2025. On May 12, 2025, he underwent transcatheter arterial chemoembolization with perfusion chemotherapy involving oxaliplatin (120 mg) and fluorouracil (650 mg). Furthermore, he had a 3-year history of hypertension, controlled with telmisartan. Based on the patient’s medication history, the suspected drugs included tislelizumab, oxaliplatin, and fluorouracil. The ALDEN (Algorithm of Drug Causality for Epidermal Necrolysis) scoring system was applied to evaluate these three drugs, yielding respective scores of 4, -1, and -1. Consequently, we made an initial diagnosis of tislelizumab-induced SJS. Regrettably, the lymphocyte transformation test was not available at our institution; additionally, the patient and his family declined to undergo this test at another facility.

**Figure 1:**
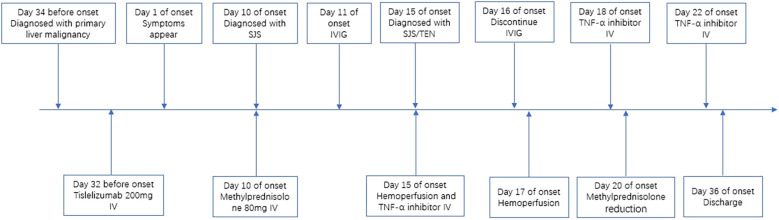
Timeline of the case report. IV, intravenous injection; IVIG, intravenous immunoglobulin.

**Figure 2:**
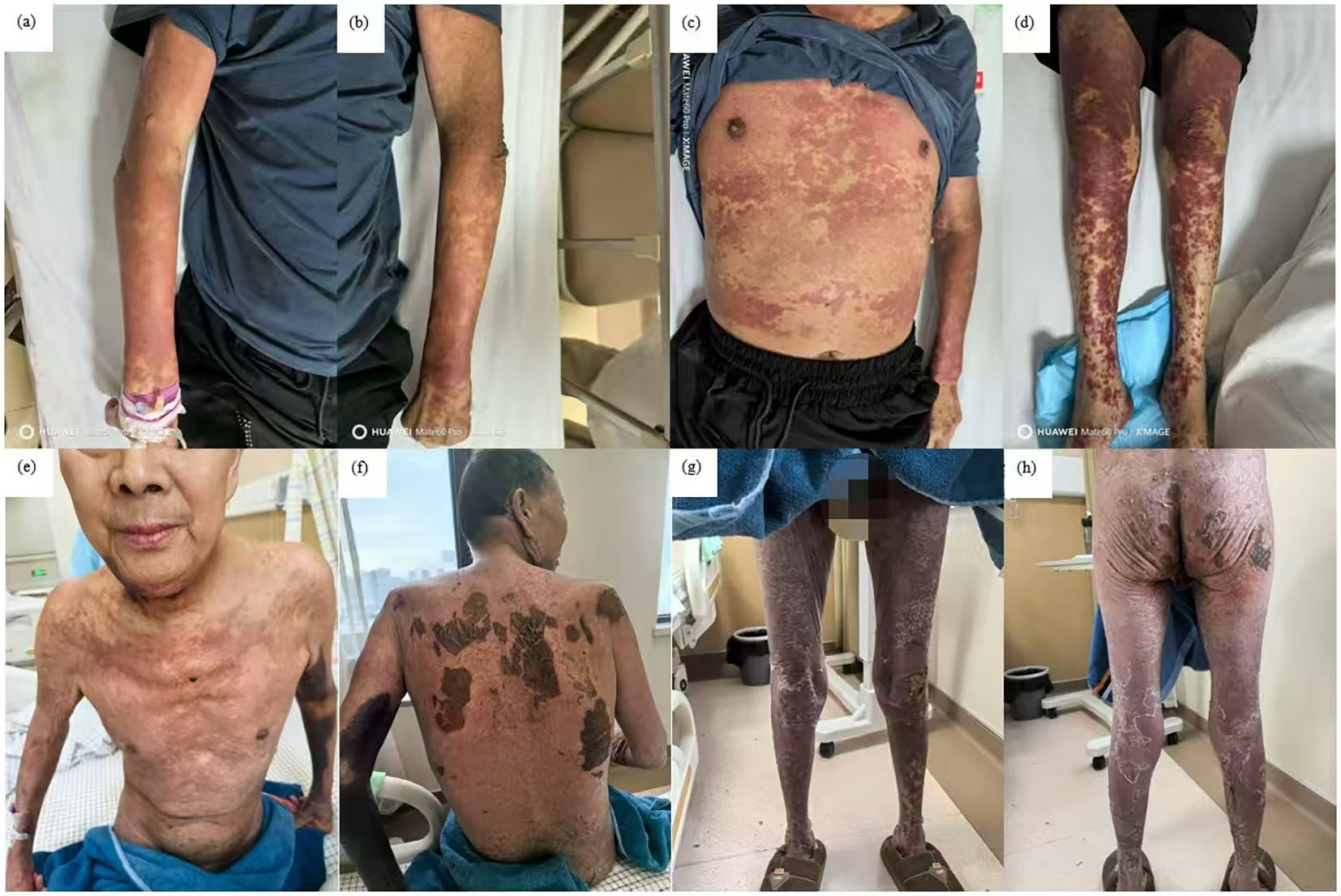
Improvement of skin lesions. Day 1: erythema all over the entire body and scattered blisters and bullae on the limbs **(a-d)**; day 16: widespread erythema gradually faded with re-epithelialization **(e-h)**.

**Figure 3:**
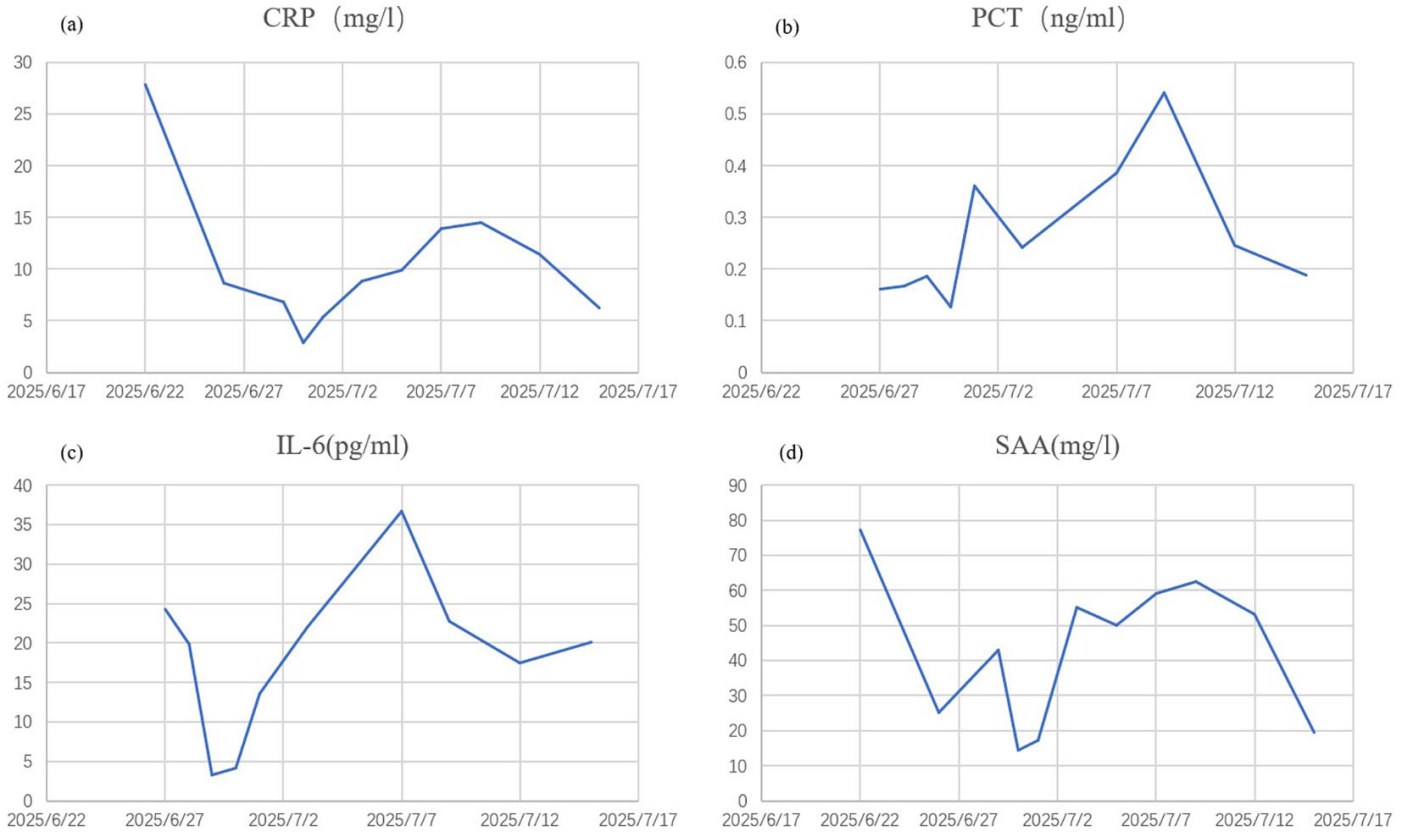
Improvement of serological indicators. CRP, C-reactive protein **(a)** PCT, procalcitonin **(b)** IL-6, interleukin-6 **(c)** SAA, serum amyloid A **(d)**.

Initially, the patient received methylprednisolone 80 mg once daily (1.38 mg/kg/day) for 5 days, in combination with intravenous immunoglobulin (IVIG) 20 g once daily (341.88 mg/kg/day) for 3 days, followed by 10 g on day 4. Adjuvant therapies included parenteral nutrition support and cefuroxime (1.5 g every 12 hours). By June 27, the patient’s skin lesions showed no significant improvement, with a notable increase in the number of vesicles and bullae. At this point, approximately 15% of his TBSA was affected by skin detachment and mucosal erosion, while over 80% of his TBSA was involved by erythema. Based on these clinical findings, we confirmed the diagnosis of SJS/TEN. At this stage, the SCORTEN was 3, corresponding to a predicted mortality rate of 35.3%. Therapy was adjusted to include hemoperfusion combined with injections of recombinant human tumor necrosis factor receptor type II-antibody fusion protein (25 mg/dose), a TNF-α inhibitor. Cefuroxime was switched to meropenem (1 g every 8 hours). One week after the treatment adjustment, the patient’s skin lesions gradually resolved, enabling the tapering of corticosteroids. The patient was hospitalized for 27 days and discharged on July 18, 2025. During hospitalization, methylprednisolone was administered as follows: 80 mg/day for 9 days, followed by 60 mg/day for 3 days, then 40 mg/day for 3 days, and finally 20 mg/day for 13 days. Hemoperfusion was performed twice, and the TNF-α inhibitor was administered three times (**Figure 1**). At discharge, almost complete re-epithelialization was observed, and the patient expressed satisfaction with the treatment outcome. The improvements in the patient’s skin lesions and serological indicators are shown in **Figures 2**, **3**, respectively.

## Discussion

We reviewed PubMed for tislelizumab-induced SJS/TEN, identifying 6 reported patients from 5 published studies (5–9). The clinical details of these 6 patients are summarized in **Table 1**. Additionally, a study focusing on tislelizumab-induced cutaneous adverse reactions reported SJS/TEN in 7 out of 13 patients, with their clinical details presented in tabular form in the original paper (10). All 13 SJS/TEN patients (9 males, 4 females; mean age 73.15 ± 7.13 years) were from China. Of these 13 patients, 8 patients had lung cancer as their primary malignancy. The treatment regimens varied, including glucocorticoid monotherapy (3/13), glucocorticoids combined with IVIG (8/13), glucocorticoids plus IVIG and cyclosporine (1/13), and glucocorticoids combined with hemoperfusion (1/13). Clinical improvement was observed in 12 patients, whereas one patient passed away due to acute coronary syndrome.

**Table 1:** Stevens-Johnson syndrome/toxic epidermal necrolysis induced by tislelizumab.

NO.	Age/Sex	Primary disease	Onset	SCORTEN	Therapy	Outcomes	Citation
1	70/M	Esophageal squamous cell carcinoma	After the 1st administration	3	Methylprednisolone + IVIG	After 2 weeks, re-epithelialization occurred.	(5)
2	73/M	Prostatic cancer & secondary lymphoid malignancies	NA	4	Methylprednisolone + Hemoperfusion	Improvement.	(6)
3	74/M	Squamous cell lung carcinoma	NA	NA	Methylprednisolone + IVIG	Improvement.	(6)
4	79/M	Esophageal squamous cell carcinoma	After the 3rd administration	4	Glucocorticoid	After 1 month, skin lesions had mostly improved.	(7)
5	70/M	Lung adenocarcinoma & prostate cancer	NA	5	Methylprednisolone + IVIG	After 31 days later, re-epithelialization was nearly complete.	(8)
6	75/M	Lung squamous cell carcinoma	After the 1st administration	NA	Methylprednisolone + IVIG	After 30 days, widespread skin had desquamated.	(9)

M, male; NA, not applicable or not specified; SCORTEN, severity-of-illness score for toxic epidermal necrolysis; IVIG, intravenous immunoglobulin.

SJS/TEN is a severe delayed-type hypersensitivity reaction mediated by T cells, in which cytotoxic CD8+ T lymphocytes are critical in its pathogenesis (3, 11). Clinical studies demonstrate elevated levels of various immune cells (e.g., CD8^+^ T cells, natural killer [NK] cells, monocytes, macrophages) and soluble mediators (e.g., TNF-α, IFN-γ, IL-6, IL-1, IL-10, perforin, granzyme) in both serum and blister fluid of SJS/TEN patients (3, 12). Keratinocyte death in SJS/TEN is mediated by a complex interplay of multiple mechanisms. Firstly, CD8^+^ T cell activation is triggered by the interaction between major histocompatibility complex (MHC) molecules on antigen-presenting cells (APCs) and T cell receptors (TCRs) on CD8^+^ T cells. Activated CD8^+^ T cells secrete TNF-α and IFN-γ, inducing keratinocytes to produce nitric oxide (NO). This NO subsequently facilitates keratinocyte death via the Fas/Fas ligand (FasL) pathway (1, 2, 11). Secondly, NK cells can mediate keratinocyte death by binding their CD94/NKG2C receptors to HLA-E molecules expressed on keratinocytes (3). Thirdly, TNF-α contributes to disease progression by upregulating epidermal expression of matrix metalloproteinase-9 (MMP-9), ultimately leading to epidermal detachment. Additionally, IFN-γ activates monocytes and dendritic cells, stimulating the production of keratinocyte-cytotoxic factors, including TNF-related apoptosis-inducing ligand (TRAIL) (11).

Post-marketing safety surveillance of tislelizumab identified 2,075 cases involving 3,795 adverse events, including 3 cases diagnosed as TEN (13). Furthermore, SJS/TEN cases induced by other PD-1 inhibitors have also been documented (14–17). Evidence indicates that programmed death-ligand 1 (PD-L1) expression is typically undetectable in normal skin. However, in cases of PD-1 inhibitor-induced SJS/TEN, PD-L1 expression is significantly upregulated in lymphocytes and keratinocytes. This upregulation may subsequently result in keratinocyte death mediated by activated CD8+ T cells (18).

Current treatment strategies for severe drug eruptions primarily include glucocorticoids, IVIG, and hemoperfusion (19). Given the role of TNF-α in the pathogenesis of severe drug eruptions, particularly SJS/TEN, some case reports have described the use of TNF-α inhibitors to treat them (19, 20). However, since malignancy and HBV infection are known contraindications for TNF-α inhibitors, their administration in patients with active malignancies or HBV infection requires careful consideration. In the present case, the patient had hepatic malignancy with lung metastases and HBV infection. Given the failure of initial therapy with glucocorticoids and IVIG to control the progressive skin lesions, after comprehensively evaluating the benefits and risks, we opted for a TNF-α inhibitor combined with hemoperfusion, which ultimately achieved a favorable outcome. This case report describes a novel therapeutic strategy for severe drug eruptions in patients with advanced malignancies.

## Data Availability

The original contributions presented in the study are included in the article/supplementary material. Further inquiries can be directed to the corresponding authors.
